# Survey of Neuromodulator Use for Optimization of Facial Scars and Blepharoplasty and Brow Lift Outcomes

**DOI:** 10.1093/asjof/ojaf005

**Published:** 2025-01-16

**Authors:** Manoj T Abraham, Solomon Husain, Anni Wong, Sunder Gidumal, Ebrahim Elahi, Ellen Marmur, Matthew DelMauro

## Abstract

**Background:**

There are many published studies that indicate neuromodulators help improve surgical outcomes and the appearance of facial scars.

**Objectives:**

To determine the prevalence of neuromodulator use as an adjunct for facial scar treatment as well as blepharoplasty and brow lift surgeries across surgical subspecialties.

**Methods:**

An anonymous electronic survey was distributed to plastic surgeons, facial plastic surgeons, oculoplastic surgeons, and dermatologic surgeons through their respective national societies. The survey assessed demographics, practice characteristics, and practice habits.

**Results:**

A total of 276 surgeons responded. Although 96.7% of respondents use neuromodulators in their practice, only 21% utilize neuromodulators for scar optimization, and 12.3% and 25.4% utilize neuromodulators for optimization of blepharoplasty and surgical brow lift outcomes, respectively.

**Conclusions:**

Although the use of neuromodulators has been shown to improve the appearance of scars and could enhance outcomes after blepharoplasty and brow lift procedures, its use among respondents was limited.

**Level of Evidence: 4 (Therapeutic):**

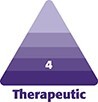

Facial scars can have profound negative sociopsychological impacts on patients—therefore, developing treatments that improve the appearance of facial scars is important.^1,2^ Sound surgical technique, laser therapies, silicone-based scar treatments, and protection from UV rays are all useful to improve the appearance of scars, but they are not always sufficient.^3^

Botulinum toxin is among the most widely known drugs in the aesthetic fields. Botulinum toxin causes temporary muscle paralysis by preventing presynaptic release of acetylcholine.^4^ When injected into and around a facial scar, botulinum toxin A (BTA) prevents contraction of the adjacent mimetic muscles and decreases tension on the wound edges, thereby reducing the risk of scar widening, scar hypertrophy, and postinflammatory hyperpigmentation.^5-8^ Two prospective double-blind randomized controlled trials demonstrated that early BTA injections following repair of facial wounds produced narrower and flatter facial surgical scars with improved cosmesis of the wound.^2,7^

In the upper face, the frontalis muscle and glabellar muscle complex are particularly strong and may contribute to suboptimal healing of nearby wounds.^9^ Repeated voluntary and involuntary movements of the orbicularis oculi muscles likewise create distracting forces that may distort wounds in the periorbital region as those wounds heal and mature.^10^ By weakening or immobilizing the upper face musculature, undesirable tension at the surgical site(s) could be reduced throughout the perioperative period, thereby improving the aesthetic and cicatricial outcomes following blepharoplasty and brow lift procedures (Figure 1).^9,11-13^

**Figure 1.**
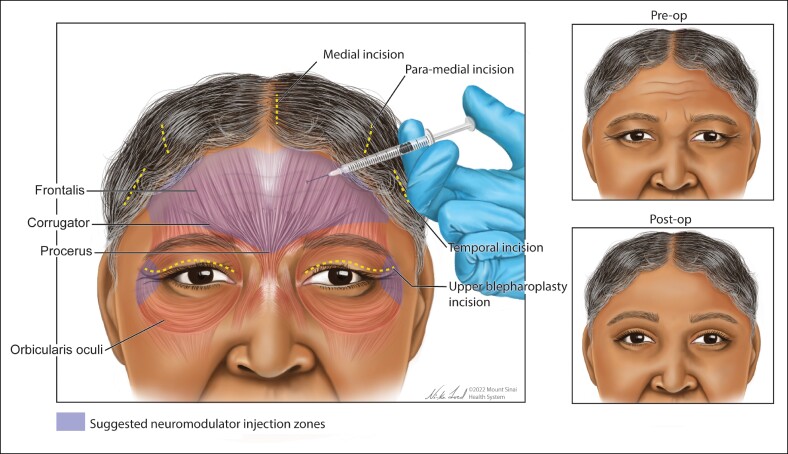
The most common muscles that are targeted for optimization of surgical results after blepharoplasty and brow lift procedures are shown. The exact pattern/location, depth, and dose of neuromodulator injections can vary greatly based on the patient and the practitioner and were not explored in this study.

Although there has been increasing interest in the use of BTA for the management of postsurgical scars and for the improvement of aesthetic outcomes after surgery, the prevalence of this intervention remains unknown. In this study, we examined the current perioperative use of BTA for facial scar optimization and for improvement of blepharoplasty and brow lift aesthetic outcomes among plastic surgery practices.

## METHODS

An anonymous electronic survey was distributed to plastic and reconstructive surgeons (PRSs), facial PRSs (FPRSs), oculoplastic and reconstructive surgeons (OPRSs), and dermatologic surgeons (DSs) in the United States. An invitation to participate in the survey was distributed through ListServe email through the American Society of Plastic Surgeons and the American Academy of Facial Plastic and Reconstructive Surgery and was posted on the online forum of the American Society of Ophthalmic Plastic and Reconstructive Surgery and on the online forum of the American Society of Dermatologic Surgery. The survey was distributed in March 2021, and responses were collected through July 2021.

The survey consisted of 18 questions regarding surgeons’ practice demographics, their use of neuromodulators, scar optimization, and familiarity with literature regarding neuromodulators for facial scar optimization. Questions comprised of a variety of response formats, including yes/no, multiple choice, and free text. The complete survey is found in the Supplemental Material with this article. The study was approved for exemption by the senior author's Institutional Review Board.

Responses were compiled and downloaded into Microsoft Excel (Microsoft Corporation, Redmond, WA). Missing answers were not included in the analyses. The data were analyzed using standard statistical methods. Significance was analyzed with χ^2^ tests, analyses of variance, and post hoc Tukey tests where significance was set at *P* < .05. A statistical analysis was performed with SPSS version 24.0 data analysis program (IBM Corp., Armonk, NY).

## RESULTS

### Demographics

A total of 276 surgeons responded to the survey: PRS, 104 of 2555 or 4.0%; FPRS, 133 of 1494 or 8.9%; OPRS, 23 of 650 or 3.5%; and DS, 23 of 3166 or 0.7%. The response rate was 3.8% of the total membership of the 4 professional societies surveyed. Demographics are shown in Table 1.

**Table 1.** Demographics of Respondents

	*n* (%)
Specialty	
Facial plastic and reconstructive surgery	133 (48.2%)
Plastic and reconstructive surgery	104 (37.7%)
Dermatologic surgery	16 (5.8%)
Oculoplastic and reconstructive surgery	23 (8.3%)
Total	276
Geographic location	
Northeast	66 (25%)
Southeast	67 (25.4%)
Midwest	43 (16.3%)
Southwest	27 (10.2%)
West	51 (19.3%)
International	10 (3.8%)
Total	264
Practice community	
Urban	89 (32.4%)
Suburban	117 (42.6%)
Rural	7 (2.5%)
Combination	62 (22.5%)
Total	275
Practice setting	
Academic institution	26 (9.5%)
Private practice	223 (81.4%)
Combination	25 (9.1%)
Total	274
Years in practice	
<5 years	28 (10.1%)
5-10 years	28 (10.1%)
11-20 years	66 (24%)
>20 years	154 (55.8%)
Total	276
Practice composition (cosmetic vs reconstructive)	
≥50% cosmetic practice	218 (80%)
<50% cosmetic practice	56 (20%)
Total	274

### Use of Neuromodulators

The majority of respondents (96.7%) use neuromodulators: 21% utilize neuromodulators for scar optimization; 12.3% use neuromodulators for the optimization of blepharoplasty outcomes; and 25.4% utilize neuromodulators for the optimization of surgical brow lift outcomes.

There were no statistically significant differences noted with the use of neuromodulator for scar, blepharoplasty, or brow lift optimization between practice specialty, geographic location, practice setting, years in practice, or practice composition. Respondents were significantly more likely to use neuromodulators in general (98.2% vs 89.7%; *P* = .002) and for optimization of blepharoplasty (14.7% vs 3.45%; *P* = .021) and brow lift surgical outcomes (29.8% vs 8.62%; *P* = .009) if their practices were predominantly (>50%) aesthetic/cosmetic vs partially (<50%) aesthetic/cosmetic. Those in private practice (26.5%) and combination practices (40%) were more likely to use neuromodulators for brow lift optimization compared with those in academic settings (3.85%, *P* = .010) as were those in practice for longer (<5 years 0%, 5-10 years 21.4%, 11-20 years 25.8%, and >20 years 30.5%, *P* = .025). A detailed summary of survey responses regarding surgeons’ use of neuromodulators is given in Supplemental Table 1.

### Neuromodulator Use for Blepharoplasty and Brow Lift Optimization

Whereas 12.3% and 25.4% of respondents use neuromodulators before blepharoplasty and brow lift procedures (respectively), 37.1% and 45.3% routinely use neuromodulators after blepharoplasty and brow lift procedures (respectively). The majority of surgeons who treat with neuromodulators before surgery do so 2 weeks before blepharoplasty (53%) and brow lift (51.4%). Repeat treatment after surgery was typically every 3 months (61.3%) or every 4 to 6 months (29%). A summary of neuromodulator use for blepharoplasty and brow lift optimization is found in Supplemental Table 2.

### Familiarity With Literature

When inquiring about respondents’ familiarity with 2 prospective double-blind randomized controlled trials, evaluating the use of neuromodulators for facial scar management, 37% and 27% of the 276 respondents were familiar with Hu et al and Ziade et al, respectively. Those who used neuromodulators specifically for scar optimization were significantly more familiar with these 2 studies (58%, *P* = .002 and 45.6%, *P* = .005). A higher percentage of those in academia was familiar with Hu et al (50% vs 34.5%, *P* = .004) and Ziade et al (53.8% vs 23.8%, *P* < .001) compared with those in private practice.

## DISCUSSION

This is the first survey to evaluate the usage of perioperative neuromodulators for facial scar optimization and for the improvement of blepharoplasty and brow lift outcomes among the 4 major plastic surgery subspecialties across the United States. Because the response rate was 3.8%, our data only provide a snapshot of those who responded to the survey. Among respondents, the majority of physicians in the fields of PRS, FPRS, OPRS, and DS use neuromodulators in some capacity in their practice (96.7%), but only a minority use neuromodulators for optimization of scars (21%) or to optimize surgical outcomes following blepharoplasty and brow lift procedures (12.3% and 25.4%, respectively). No significant difference was identified between the different subspecialties.

To date, there are many studies that support the use of neuromodulators preoperatively to enhance surgical aesthetic outcomes. Improved cosmesis of neuromodulator-treated wound repairs has been demonstrated in the forehead,^5,8,12,14^ periorbit,^10,15^ and perioral region.^16-18^ Moreover, numerous studies demonstrate improvement of facial scars with early injection of neuromodulators.^1,2,7,12,14,17,19^ The incongruence between the low percentage of practitioners using neuromodulators for these indications in our study and the abundant literature available on this subject points to a gap between available knowledge and contemporary practice.

Although there is only a descriptive report of neuromodulators use for surgical optimization of brow lift by Nassif^11^ with no studies offering comparative evidence of benefit, approximately a quarter of the respondents endorsed the use of perioperative neuromodulators for brow lift procedures. It has been established that neuromodulator treatment can chemically induce a brow lift.^20,21^ However, the adoption of this technique in the perioperative setting despite a lack of solid evidence in the medical literature is likely the result of anecdotal reports. These practitioners argue that relaxing the brow depressors before brow lifting minimizes downward muscle action on the tail of the brow during the healing process, improving the surgical outcome.^11^ Sweis et al advocate for the preoperative use of neuromodulators in the frontalis muscle in those patients with frontalis hyperactivity and advocate against preoperative use of neuromodulators on brow depressors, which the authors state permits a more accurate preoperative assessment of the brow–lid complex by minimizing confounding vectors of tension.^13^ A known risk of brow lift procedures is injury to the frontal branch of the facial nerve, and treatment of the frontalis muscle with neuromodulator will make it more difficult to evaluate the function of the nerve in the postoperative setting. Similar to Nassif, Sweis et al recommend neutralization of brow depressor muscles with neuromodulators during the postoperative period.

In our study, only 12% of surgeons have adopted the use of perioperative neuromodulators for blepharoplasty. Although some studies have shown that perioperative neuromodulator use does improve blepharoplasty scars^12^ as well as wound healing after eyelid reconstruction,^15^ the low rate of adoption could certainly be due to the limited number of descriptive and comparative studies on the subject. Additionally, equivocal anecdotal reports and other confounding factors (such as differences in skin type) may also play a role.

As with any research based on survey data, the present study is limited by its design and is subject to response bias. The present study is also subject to sampling bias, as responses may be limited to those who are particularly interested in the subject and opted to open the survey email invitation or access the society's forum when the survey posted.

The discordance between the low frequency of neuromodulator use for scar optimization despite the abundant literature regarding its benefits points to the need for better education across all of the aesthetic specialties and potentially the need for standardized clinical practice guidelines to facilitate universal adoption of neuromodulator use for scar optimization. Given the paucity of literature investigating surgical outcomes of blepharoplasties and brow lifts with and without the use of neuromodulators, as well as the timing of neuromodulator use, additional studies would be beneficial.

## CONCLUSIONS

The use of neuromodulators for facial scar optimization and for the optimization of blepharoplasty and brow lift outcomes remains limited among the 4 major plastic surgery specialties surveyed in our study. Less than half of the respondents are familiar with 2 definitive prospective double-blind randomized controlled trials regarding the use of neuromodulators for facial scar optimization. This incongruence between available evidence and clinical practice indicates the need for better education and standardized treatment algorithms. On the other hand, about a quarter of respondents use neuromodulators for optimization of brow lift outcomes despite limited literature describing the benefit of perioperative neuromodulators in blepharoplasty and brow lift procedures. Additional controlled studies are in process and will be useful to better elucidate this topic.

## Supplementary Material

ojaf005_Supplementary_Data

## References

[1] Kerwin LY , El Tal AK, Stiff MA, Fakhouri TM. Scar prevention and remodeling: a review of the medical, surgical, topical and light treatment approaches. Int J Dermatol. 2014;53:922–936. doi: 10.1111/ijd.12436 24697346

[2] Hu L , Zou Y, Chang SJ, et al Effects of botulinum toxin on improving facial surgical scars: a prospective, split-scar, double-blind, randomized controlled trial. Plast Reconstr Surg. 2018;141:646–650. doi: 10.1097/PRS.0000000000004110 29481395

[3] Son D , Harijan A. Overview of surgical scar prevention and management. J Korean Med Sci. 2014;29:751–757. doi: 10.3346/jkms.2014.29.6.751 24932073 PMC4055805

[4] Dressler D , Adib Saberi F. Botulinum toxin: mechanisms of action. Eur Neurol. 2005;53:3–9. doi: 10.1159/000083259 15650306

[5] Gassner HG , Mueller-Vogt U, Strutz J, Kuehnel T. Nasal tip recontouring in primary rhinoplasty: the endonasal complete release approach. JAMA Facial Plast Surg. 2013;15:11–16. doi: 10.1001/jamafacial.2013.223 23165886

[6] Jablonka EM , Sherris DA, Gassner HG. Botulinum toxin to minimize facial scarring. Facial Plast Surg. 2012;28:525–535. doi: 10.1055/s-0032-1325641 23027220

[7] Ziade M , Domergue S, Batifol D, et al Use of botulinum toxin type A to improve treatment of facial wounds: a prospective randomised study. J Plast Reconstr Aesthet Surg. 2013;66:209–214. doi: 10.1016/j.bjps.2012.09.012 23102873

[8] Lee SH , Min HJ, Kim YW, Cheon YW. The efficacy and safety of early postoperative botulinum toxin A injection for facial scars. Aesthetic Plast Surg. 2018;42:530–537. doi: 10.1007/s00266-017-1008-7 29214336

[9] Lin MJ , Dubin DP, Khorasani H. Botulinum toxin for paramedian interpolated forehead flaps. J Cutan Aesthet Surg. 2020;13:170–172. doi: 10.4103/JCAS.JCAS_56_19 32792781 PMC7394119

[10] Huang YL , Wallace CG, Hsiao YC, et al Botulinum toxin to improve lower blepharoplasty scar: a double-blinded, randomized, vehicle-controlled clinical trial. Aesthet Surg J. 2021;41:1003–1010. doi: 10.1093/asj/sjab024 34128526

[11] Nassif PS . Endoscopic brow-lift with deep temporal fixation only (DTFO). Facial Plast Surg Clin North Am. 2006;14:203–211. doi: 10.1016/j.fsc.2006.04.004 16908387

[12] Zelken J , Yang SY, Chang CS, et al Donor site aesthetic enhancement with preoperative botulinum toxin in forehead flap nasal reconstruction. Ann Plast Surg. 2016;77:535–538. doi: 10.1097/SAP.0000000000000625 26418784

[13] Sweis IE , Hwang L, Cohen M. Preoperative use of neuromodulators to optimize surgical outcomes in upper blepharoplasty and brow lift. Aesthet Surg J. 2018;38:941–948. doi: 10.1093/asj/sjy049 29474688

[14] Kim SH , Lee SJ, Lee JW, Jeong HS, Suh IS. Clinical trial to evaluate the efficacy of botulinum toxin type A injection for reducing scars in patients with forehead laceration: a double-blinded, randomized controlled study. Medicine (Baltimore). 2019;98:e16952. doi: 10.1097/MD.0000000000016952 31441893 PMC6716761

[15] Choi JC , Lucarelli MJ, Shore JW. Use of botulinum A toxin in patients at risk of wound complications following eyelid reconstruction. Ophthalmic Plast Reconstr Surg. 1997;13:259–264. doi: 10.1097/00002341-199712000-00006 9430303

[16] Bartkowska P , Roszak J, Ostrowski H, Komisarek O. Botulinum toxin type A as a novel method of preventing cleft lip scar hypertrophy—a literature review. J Cosmet Dermatol. 2020;19:2188–2193. doi: 10.1111/jocd.13614 32654297

[17] Chang CS , Wallace CG, Hsiao YC, Chang CJ, Chen PKT. Botulinum toxin to improve results in cleft lip repair. Plast Reconstr Surg. 2014;134:511–516. doi: 10.1097/PRS.0000000000000416 25158709

[18] Tollefson TT , Senders CM, Sykes JM, Byorth PJ. Botulinum toxin to improve results in cleft lip repair. Arch Facial Plast Surg. 2006;8:221–222. doi: 10.1001/archfaci.8.3.221 16702537

[19] Wang D , Qu J, Jiang H, Jiang Y. The safety and efficacy of botulinum toxin for management of scars: a systematic review with meta-analysis and trial sequential analysis. Toxicon. 2019;166:24–33. doi: 10.1016/j.toxicon.2019.04.018 31047933

[20] Ahn MS , Catten M, Maas C. Temporal brow lift using botulinum toxin A. Plast Reconstr Surg. 2000;105:1129–1135. doi: 10.1097/00006534-200003000-00046 10724275

[21] Frankel AS , Kamer FM. Chemical browlift. Arch Otolaryngol Head Neck Surg. 1998;124:321–323. doi: 10.1001/archotol.124.3.321 9525518

