# Supplementary material for: Survey of Neuromodulator Use for Optimization of Facial Scars and Blepharoplasty and Brow Lift Outcomes
Source: Aesthet Surg J Open Forum. 2025 Jan 16;7:ojaf005. doi: 10.1093/asjof/ojaf005 (PMC11842229; doi:10.1093/asjof/ojaf005)
Supplement: ojaf005_Supplementary_Data [file ojaf005_supplementary_data.zip › Supplemental Table 1.docx]

**Supplemental Content 1: Summary of Neuromodulator (NM) Use**

|  | **Use of NM** | **Use of NM for scar optimization** | **Use of NM for blepharoplasty optimization** | **Use of NM for brow lift optimization** | **Familiar with Hu et al publication** | **Familiar with Ziade et al publication** |
| --- | --- | --- | --- | --- | --- | --- |
| **Summary** |  |  |  |  |  |  |
| Yes | 266 (96.7%) | 57 (21%) | 34 (12.3%) | 70 (25.4%) |  |  |
|  |  |  |  |  |  |  |
| **Specialty** |  |  |  |  |  |  |
| FPRS (N=134) | 132 (98.5%) | 35 (26.1%) | 18 (13.4%) | 44 (32.8%) |  |  |
| PRS (N =103) | 101 (98%) | 15 (14.6%) | 14 (13.6%) | 21 (20.4%) |  |  |
| DS (N=23) | 17 (74%) | 5 (21.7%) | 1 (4.34%) | 1 (4.34%) |  |  |
| OPRS(N=16) | 16 (100%) | 2 (12.5%) | 1 (6.25%) | 4 (25%) |  |  |
|  | *p* <.001^a^ | *p*=.142 | *p*=.531 | *p*=.014^a^ |  |  |
|  |  |  |  |  |  |  |
| **Geographic location** |  |  |  |  |  |  |
| Northeast (N= 66) | 64 (97%) | 19 (28.8%) | 10 (15.2%) | 18 (27.3%) |  |  |
| Southeast (N=67) | 65 (97%) | 10 (15%) | 5 (7.46%) | 18 (26.9%) |  |  |
| Midwest (N=43) | 41 (95.3%) | 8 (18.6%) | 5 (11.6%) | 7 (16.3%) |  |  |
| Southwest (N=27) | 25 (92.6%) | 2 (7.41%) | 2 (7.41%) | 3 (1.11%) |  |  |
| West (N=51) | 49 (96.1%) | 10 (19.6%) | 10 (19.6%) | 18 (35.3%) |  |  |
| International (N=10) | 10 (100%) | 4 (40%) | 2 (20%) | 3 (30%) |  |  |
|  | *p*=.874 | *p*=.136 | *p*=0.291 | *p*=.105 |  |  |
| **Practice Community** |  |  |  |  |  |  |
| Urban (N=89) | 86 (96.6%) | 19 (21.3%) | 13 (21.3%) | 27 (30.3%) |  |  |
| Suburban (N=117) | 112 (95.7%) | 22 (18.8%) | 14 (12%) | 22 (18.8%) |  |  |
| Rural (N=7) | 7 (100%) | 1 (14.3%) | 1 (14.3%) | 2 (28.5%) |  |  |
| Combination (N=63) | 60 (95.2%) | 15 (23.8%) | 6 (9.52%) | 19 (30.2%) |  |  |
|  | *p* =.679 | *p* =.843 | *p*=.774 | *p*=.201 |  |  |
| **Practice Setting** |  |  |  |  |  |  |
| Academic (N=26) | 23 (88.55%) | 5 (19.2%) | 1 (3.85%) | 1 (3.85%) | 13 (50%) | 14 (53.8%) |
| Private (N=223) | 217 (97.3%) | 45 (20.2%) | 27 (12.1%) | 59 (26.5%) | 77 (34.5%) | 53 (23.8%) |
| Combination (N=25) | 24 (96%) | 7 (28%) | 6 (24%) | 10 (40%) | 11 (44%) | 7 (28%) |
|  | *p*=.765 | *p*=.662 | *p*=.088 | *p*= .010^a^ | *p= 0.004* | *p* <.001 |
| **Years in practice** |  |  |  |  |  |  |
| < 5 years (N=28) | 27 (96.4%) | 6 (21.4%) | 0 | 0 |  |  |
| 5-10 years (N=28) | 27 (96.4%) | 5 (17.9%) | 4 (14.3%) | 6 (21.4%) |  |  |
| 11-20 years (N=66) | 62 (94%) | 19 (28.8%) | 7 (10.6%) | 17 (25.8%) |  |  |
| > 20 years (N=154) | 150 (97%) | 27 (17.5%) | 23 (15%) | 47 (30.5%) |  |  |
|  | *p*=.662 | *p*=.293 | *p*= .372 | *p*=.025^a^ |  |  |
| **Practice composition** |  |  |  |  |  |  |
| ≥50% cosmetic practice (N=218) | 214 (98.2%) | 48 (22%) | 32 (14.7%) | 65 (29.8%) |  |  |
| <50% cosmetic practice (N=58) | 52 (89.7%) | 9 (15.5%) | 2 (3.45%) | 5 (8.62%) |  |  |
|  | *p*=.002^a^ | *p*=.277 | *p*=.021^a^ | *p*=.009^a^ |  |  |

^a^ statistical significance at *p* <0.05; FPRS: Facial Plastic and Reconstructive Surgery, PRS: Plastic and Reconstructive Surgery, DS: Dermatologic Surgery, OPRS: Oculoplastic and Reconstructive Surgery
