# Supplementary material for: Survey of Neuromodulator Use for Optimization of Facial Scars and Blepharoplasty and Brow Lift Outcomes
Source: Aesthet Surg J Open Forum. 2025 Jan 16;7:ojaf005. doi: 10.1093/asjof/ojaf005 (PMC11842229; doi:10.1093/asjof/ojaf005)
Supplement: ojaf005_Supplementary_Data [file ojaf005_supplementary_data.zip › Supplemental Table 2.docx]

**Supplemental Content 2: Neuromodulator use for blepharoplasty and brow lift optimization**

|  | **N (%)** | **Avg units used for frontalis (range)**^a^ | **Avg units used for procerus & corrugators (range)**^a^ | **Avg units used for orbicularis oculi (range)**^a^ |
| --- | --- | --- | --- | --- |
| **NM Use for blepharoplasty optimization (N=39)** |  |  |  |  |
| Botox^®^ | 31 (79.5%) |  |  |  |
| Dysport^®^ | 10 (25.6%) |  |  |  |
| Jeuveau^®^ | 6 (15.4%) |  |  |  |
| Xeomin^®^ | 8 (20.5%) |  |  |  |
| Vistabel^®^ | 1 (2.6%) |  |  |  |
|  |  | 16 units (4-40) | 18 units (4-25) | 12 units (4-20) |
|  |  |  |  |  |
| **NM Use for brow lift optimization (N=75)** |  |  |  |  |
| Botox^®^ | 62 (82.7%) |  |  |  |
| Dysport^®^ | 17 (22.7%) |  |  |  |
| Jeuveau^®^ | 7 (9.3%) |  |  |  |
| Xeomin^®^ | 3 (4%) |  |  |  |
|  |  | 16 units (4-40) | 19 units (4-30) | 10.5 units (5-25) |
| ^a^ All NM dosage units converted to Botox^®^ equivalent dosage | | | | |
|  |  | **Blepharoplasty** | **Brow lift procedure** |  |
| **Routine use of maintenance NM treatment following surgery (N=276)** |  |  |  |  |
| Yes |  | 91 (33%) | 111 (40.2%) |  |
| No |  | 27 (9.78%) | 29 (10.5%) |  |
| Not Applicable |  | 127 (46%) | 105 (38%) |  |
| No Response |  | 31 (11.2%) | 31 (11.2%) |  |
|  |  |  |  |  |
| **Frequency of NM maintenance treatment (N=93)** |  |  |  |  |
| Every 3 months | 57 (61.3%) |  |  |  |
| Every 4- 6 months | 27 (29%) |  |  |  |
| As needed/ patient preference | 9 (9.68%) |  |  |  |
|  |  |  |  |  |
| **Time prior to surgery with treatment with NM** |  | **N = 34** | **N = 70** |  |
| 1 week prior surgery |  | 6 (17.6%) | 20 (28.6%) |  |
| 2 weeks prior surgery |  | 18 (53%) | 36 (51.4%) |  |
| > 2 weeks prior surgery |  | 10 (29.4%) | 14 (20%) |  |
